# Silylated Zeolites With Enhanced Hydrothermal Stability for the Aqueous-Phase Hydrogenation of Levulinic Acid to γ-Valerolactone

**DOI:** 10.3389/fchem.2018.00143

**Published:** 2018-05-16

**Authors:** Hue-Tong Vu, Florian M. Harth, Nicole Wilde

**Affiliations:** Institute of Chemical Technology, Universität Leipzig, Leipzig, Germany

**Keywords:** hydrothermal stability, biomass, hydrogenation, levulinic acid, formic acid, γ-valerolactone, silylation, zeolite Y

## Abstract

A systematic silylation approach using mono-, di-, and trichlorosilanes with different alkyl chain lengths was employed to enhance the hydrothermal stability of zeolite Y. DRIFT spectra of the silylated zeolites indicate that the attachment of the silanes takes place at surface silanol groups. Regarding hydrothermal stability under aqueous-phase processing (APP) conditions, i.e., pH ≈ 2, 473 K and autogenous pressure, the selective silylation of the zeolite surface using monochlorosilanes has no considerable influence. By using trichlorosilanes, the hydrothermal stability of zeolite Y can be improved significantly as proven by a stability test in an aqueous solution of 0.2 M levulinic acid (LA) and 0.6 M formic acid (FA) at 473 K. However, the silylation with trichlorosilanes results in a significant loss of total specific pore volume and total specific surface area, e.g., 0.35 cm^3^ g^−1^ and 507 m^2^ g^−1^ for the silylated zeolite Y functionalized with n-octadecyltrichlorosilane compared to 0.51 cm^3^ g^−1^ and 788 m^2^ g^−1^ for the parent zeolite Y. The hydrogenation of LA to γ-valerolactone (GVL) was conducted over 3 wt.-% Pt on zeolite Y (3PtY) silylated with either n-octadecyltrichlorosilane or methyltrichlorosilane using different reducing agents, e.g., FA or H_2_. While in the stability test an enhanced hydrothermal stability was found for zeolite Y silylated with n-octadecyltrichlorosilane, its stability in the hydrogenation of LA was far less pronounced. Only by applying an excess amount of methyltrichlorosilane, i.e., 10 mmol per 1 g of zeolite Y, presumably resulting in a high degree of polymerization among the silanes, a recognizable improvement of the stability of the 3 PtY catalyst could be achieved. Nonetheless, the pore blockage found for zeolite Y silylated with an excess amount of methyltrichlorosilane was reflected in a drastically lower GVL yield at 493 K using FA as reducing agent, i.e., 12 vs. 34% for 3PtY after 24 h.

## Introduction

Today's global economies heavily rely on the utilization of fossil resources. The most predominant side effect of this dependence is the emission of greenhouse gases resulting in global warming and climate changes. Additionally, the uncertain supply of crude oil also drives the accelerated search for sustainable substitutes in order to reduce the strong reliance on fossil resources. In this regard, biomass has been extensively studied as a renewable resource for the production of chemicals and fuels due to its ubiquity (Alonso et al., 2010).

Lignocellulose, i.e., non-edible plant biomass, is the most abundant class of biomass and is commonly produced as a waste by-product in the agricultural and forestry industry. Aqueous-phase processing (APP) is a selective and comparatively mild approach to utilize lignocellulosic biomass via hydrolysis and subsequent heterogeneously catalyzed upgrading. In this respect, the highly oxygenated macromolecules as present in lignocellulosic biomass are depolymerized and converted to a number of platform chemicals, including hydroxymethylfurfural (HMF), levulinic acid (LA), 2-methyltetrahydrofuran (Alonso et al., 2013), levulinate esters (Sun and Cheng, 2002), δ-aminolevulinic acid (Chheda et al., 2007), or γ-valerolactone (GVL) (Cortright et al., 2002; Huber et al., 2004; Gallezot, 2012). Of these, GVL has attracted high interest as a potential fuel additive, a “green solvent,” an intermediate for the manufacture of chemicals (Alonso et al., 2013) and a new class of biofuels named “valeric biofuels” (Lange et al., 2010). The production of GVL entails the hydrogenation of LA. LA can be selectively produced via acid hydrolysis of lignocellulosic biomass, which results in a high-water content mixture containing formic acid (FA) as by-product with an equimolar amount (Kamm et al., 2005). Therefore, the use of the as-synthesized aqueous mixture of LA and FA allows economically efficient production of GVL since no subsequent separation is required for further upgrading. In most studies, molecular hydrogen is used as a reducing agent for hydrogenation of LA (Mehdi et al., 2008; Deng et al., 2009, 2010; Geilen et al., 2010; Li et al., 2012; Wright and Palkovits, 2012; Delhomme et al., 2013; Luo et al., 2013; Abdelrahman et al., 2015; Huang et al., 2015). However, the direct utilization of FA is a promising approach to avoid the application of external hydrogen supply. Possible reaction pathways proposed for the hydrogenation of LA toward GVL involve two steps, hydrogenation and acid-catalyzed dehydration (Abdelrahman et al., 2014). Hence, bifunctional catalysts, containing both acid sites, e.g., zeolites, SiO_2_-Al_2_O_3_, and metal sites, e.g., Pt, Pd, Ru, to catalyze dehydration and hydrogenation reactions, respectively, are required (Scheme S.1). In this regard, zeolites appear to be the promising materials for the preparation of the bifunctional catalyst due to the adjustable acidic properties, i.e., strength and density of both Brønsted and Lewis acid sites (Li et al., 2012; Ennaert et al., 2016). Owing to the comparatively large specific surface area, zeolites can also act as support materials for well dispersed metal particles.

Even though zeolites and especially ultra-stable zeolite Y (USY) are known for their tolerance to steam, their stability in aqueous-phase processes employing hot liquid water is less pronounced (Galadima and Muraza, 2017). Under this severe environment, zeolites suffer framework collapse, which was shown to be facilitated by the presence of silanol defects (Ravenelle et al., 2010; Ennaert et al., 2015). Zhang et al. (2015) demonstrated the vital role of the density of Si-OH groups in determining the stability of zeolites in hot liquid water at 473 K. Additionally, the silylation with ethyltrichlorosilane was found to significantly improve the stability of USY. This method both decreases the surface silanol density and increases the surface hydrophobicity, which prevents attack by water. Based on a similar concept, Prodinger et al. (2016) demonstrated the stability-enhancing effect of the selective silylation using chlorotrimethylsilane on retaining the framework of BEA zeolites after 48 h at 433 K in hot liquid water. However, systematic studies on the stability of zeolites and stabilization strategies via silylation have not been reported in acidic aqueous solutions, typically at pH ≈ 2, under APP conditions, i.e., 473 K.

Thus, in the present study, a systematic silylation approach using mono-, di-, and trichlorosilanes with different alkyl chain lengths, i.e., C1, C3, and C18, was employed to enhance the hydrothermal stability of zeolite Y. The hydrothermal stability of the resulting silylated zeolites was investigated in an aqueous acidic solution containing 0.2 M LA and 0.6 M FA under APP conditions. For Pt catalysts on selected silylated zeolites the activity and selectivity toward GVL were assessed in the aqueous-phase *in-situ* hydrogenation of LA using different reducing agents, i.e., FA or H_2_.

## Materials and methods

### Materials

Zeolite Y (CBV 720, H^+^ form, *n*_Si_/*n*_Al_ = 15) was supplied by Zeolyst. Levulinic acid (LA) (≥98%) was provided by Merck Schuchardt OHG. Formic acid (FA) (99–100%) and GVL (99%) were obtained from Sigma–Aldrich. Toluene (100%) and ethanol (99.8%) were purchased from VWR International S.A.S. Tetraammine platinum (II) nitrate [Pt(NH_3_)_4_(NO_3_)_2_, 99.99%] was purchased from Alfa Aesar. The silylating agents are listed in Table S.1. All chemicals were used as received without further purification.

### Catalyst preparation

#### Surface modification via silylation using organosilanes

The silylated zeolites were prepared via a procedure developed by Zapata et al. (2013). Accordingly, 2 g of zeolite Y was dried in static air at 373 K overnight and subsequently dispersed in 40 cm^3^ toluene using an ultrasonic bath. The silylating agent (0.5 mmol organosilane per 1 g of zeolite, unless noted) was dissolved in 50 cm^3^ toluene and added to the zeolite suspension. The mixture was stirred for 24 h at room temperature before the zeolite was collected, washed with ethanol and dried overnight at 373 K in static air. A variety of organosilanes comprising mono-, di-, trichlorosilanes with different alkyl chain lengths were applied (cf. Table S.1). The obtained zeolites were labeled as *x*Cl*y*C*z* with *x* being the number of chlorine atoms in the respective silane molecule (*x* = 1, 2, 3), *y* being the number of carbon atoms in the alkylsilyl group (*y* = 1, 3, 8, 18), and *z* being the organosilane/zeolite ratio (*z* = 0.5, 10 mmol per 1 g of zeolite) (cf. Table S.1).

#### Silylation of Pt/zeolite Y catalyst

In the first step, Pt/zeolite Y catalyst was prepared via incipient wetness impregnation. In a typical experiment, to obtain an aimed loading of 3 wt.-% of Pt, a solution of 0.061 g of tetraammine platinum (II) nitrate in 0.75 cm^3^ of deionized water was added dropwise to 1 g of parent zeolite Y. The resulting solid was then dried in static air at 373 K for 12 h and subsequently calcined for 4 h at 723 K with a heating rate of 5 K min^−1^ in static air. The calcined catalyst (600 mg) was reduced at 673 K under a flow of H_2_ (2.0 cm^3^ min^−1^) in N_2_ (8.0 cm^3^ min^−1^) for 4 h.

Subsequent silylation of the Pt/zeolite Y catalyst using n-octadecyltrichlorosilane and methyltrichlorosilane with the ratio of organosilanes to zeolite being 0.5 and 10 mmol g^−1^ respectively yielded the modified catalysts (cf. section Surface Modification via Silylation Using Organosilanes). Prior to the catalytic experiment, a second reduction for these silylated catalysts was conducted at 473 K under a flow of H_2_ (2.0 cm^3^ min^−1^) in N_2_ (8.0 cm^3^ min^−1^) for 4 h.

### Characterization

The parent zeolite Y and silylated zeolite Y, as well as Pt/Y (3PtY) and silylated Pt/Y (3PtYxClyCz) were characterized by diffuse reflectance infrared Fourier transform spectroscopy (DRIFTS), N_2_ physisorption, elemental analysis via optical emission spectroscopy with inductively coupled plasma (ICP-OES), and powder X-ray diffraction (XRD).

DRIFTS was carried out in a Bruker Vector 22 FTIR solid phase spectrometer equipped with a heated DRIFTS cell with a ZnSe window. Prior to the measurement, the samples were dried at 473 K for 30 min and the spectra were taken at 373 K in a N_2_ flow rate of 100 cm^3^ min^−1^. Spectra were recorded under a N_2_ atmosphere at 373 K, in the range of 800–4,000 cm^−1^, by addition of 100 scans and with a nominal resolution of 4 cm^−1^.

A micromeritics ASAP2010—physisorption analyzer was used to record N_2_ physisorption isotherms. The samples were evacuated at 523 K under vacuum pressure of 3 × 10^−11^ MPa for 6 h prior to the measurements. The isotherms were taken at 77 K. The total specific surface area (A_BET_) was determined by the Brunauer-Emmett-Teller (BET) model. The total specific pore volume (V_total_) was estimated from the N_2_ uptake at a relative pressure (p ${\text{p}}_{\text{o}}^{-1}$) of 0.99. The specific micropore surface area and specific micropore volume were calculated using the t-plot model.

The Pt content was determined by optical emission spectroscopy with inductively coupled plasma (ICP-OES) using a PerkinElmer Optima 8000. Prior to the analysis, the samples were dissolved in 2.0 cm^3^ HF, 3.0 cm^3^ HNO_3_, and 3.0 cm^3^ HCl and diluted to obtain aqueous solutions which also contained 12.0 cm^3^ H_3_BO_4_ for complexation of excessive HF.

Powder XRD patterns were recorded at room temperature using a Siemens, D5000 diffractometer. The diffracted intensity of Cu-Kα radiation (λ = 0.154 nm) was measured in the range of 2θ between 4° and 90°, with a step size of 0.005° and a counting time of 0.2 second for phase identification.

### Stability test

Stability tests were conducted with zeolite Y and silylated zeolite Y. The reactant solution used in the stability test was the same used for the catalytic experiments (see below), which was a 5 wt.-% aqueous solution of LA (0.2 M) and FA (0.6 M). In a stainless steel autoclave with a 60 cm^3^ polytetrafluoroethylene (PTFE) liner, 1 g of the zeolite sample, dried at 373 K for 12 h prior to the experiment, was added to 40 cm^3^ of the reactant solution. The sealed autoclave was kept in the oven at 473 K for 24 h. Afterwards, the parent zeolite Y was separated via centrifugation, and subsequently washed three times with 30 cm^3^ of deionized water. Silylated samples were separated by filtration, and subsequently washed three times with 30 cm^3^ of ethanol. Finally, the samples were dried overnight at 373 K in static air.

### Aqueous-phase hydrogenation of levulinic acid

Reactions were carried out in a 300 cm^3^ stainless steel batch reactor (Model # 4560, *Parr Instruments Company*) with a head stirrer, a heater, as well as an external monitor (Model # 4848, *Parr Instruments Company*) for temperature, pressure and stirring speed. For each catalytic experiment, 0.50 g of the pre-reduced catalyst and 125 cm^3^ of an aqueous solution containing LA (0.2 M) were loaded into the reactor. The reactor was sealed, purged with a flow of N_2_ (4.0 MPa) for 15 min, heated up to 393 or 493 K and kept at that temperature for 24 h while stirring at 700 min^−1^. Either FA or H_2_ was applied as the reducing agent. In the first case, FA was added directly to the starting reactant solution, giving a 5 wt.-% aqueous solution of LA (0.2 M) and FA (0.6 M) and the reaction ran at autogenous pressure. In the latter case, an excess amount of gaseous H_2_ (2.5 MPa) was applied to the reactor when the desired temperature was reached, typically after 30 min.

Liquid samples were withdrawn at the start (when the desired temperature was reached, typically after 30 min) and after 24 h. The withdrawn samples were filtered and diluted by a volume factor of 5 in triple deionized water. A high-performance liquid chromatography (HPLC) system (Prominence-HPLC, Shimadzu, Kyoto, Japan) equipped with a photo-diode array detector and a Macherey-Nagel Nucleodur PolarTec column (4.6 × 250 mm) was used for the quantification of FA, LA, and GVL. An aqueous solution of 5 M H_2_SO_4_ was used as the mobile phase at a flow rate of 0.8 cm^3^ min^−1^ and the column was operated at 313 K. The substances were quantified using chromatograms at the wavelength of 210 nm. Retention times for FA, LA, and GVL were determined using commercial FA, LA, and GVL.

The conversion of FA (*X*_*FA*_), LA (*X*_*LA*_), and the yield of GVL (*Y*_*GVL*_) were calculated from the concentration of the compounds determined via external calibration of the respective integrated peak area.

(1) $$\begin{array}{l} X_{FA}=\frac{{\text{C}}_{0,\text{FA}}-\;{\text{C}}_{\text{t},\text{ FA}}}{{\text{C}}_{0,\text{ FA}}}\;\times \;100\;\% \end{array}$$

(2) $$X_{LA}\;=\frac{{\text{C}}_{0,\text{LA}}-\;{\text{C}}_{\text{t},\text{ LA}}}{{\text{C}}_{0,\text{ LA}}}\;\times \;100\;\%$$

(3) $$Y_{GVL}=\frac{{\text{C}}_{\text{t},\text{ GVL}}}{{\text{C}}_{\text{theoretical},\text{ GVL}}}\;\times \;100\;\%$$

C_o,FA,LA_: initial FA, LA concentration, C_t, FA, LA, GVL_: FA, LA, GVL concentration at specific reaction time and C_theoretical, GVL_ represents the stoichiometric calculated yield of GVL.

After the reaction, the reactor was cooled to room temperature, and the catalyst was removed from the reaction mixture by centrifugation, washed three times with 30 cm^3^ deionized water and dried at 373 K for 12 h.

## Results and discussion

### Impact of attached organosilanes on textural and structural properties of zeolite Y

**Figure 1:**
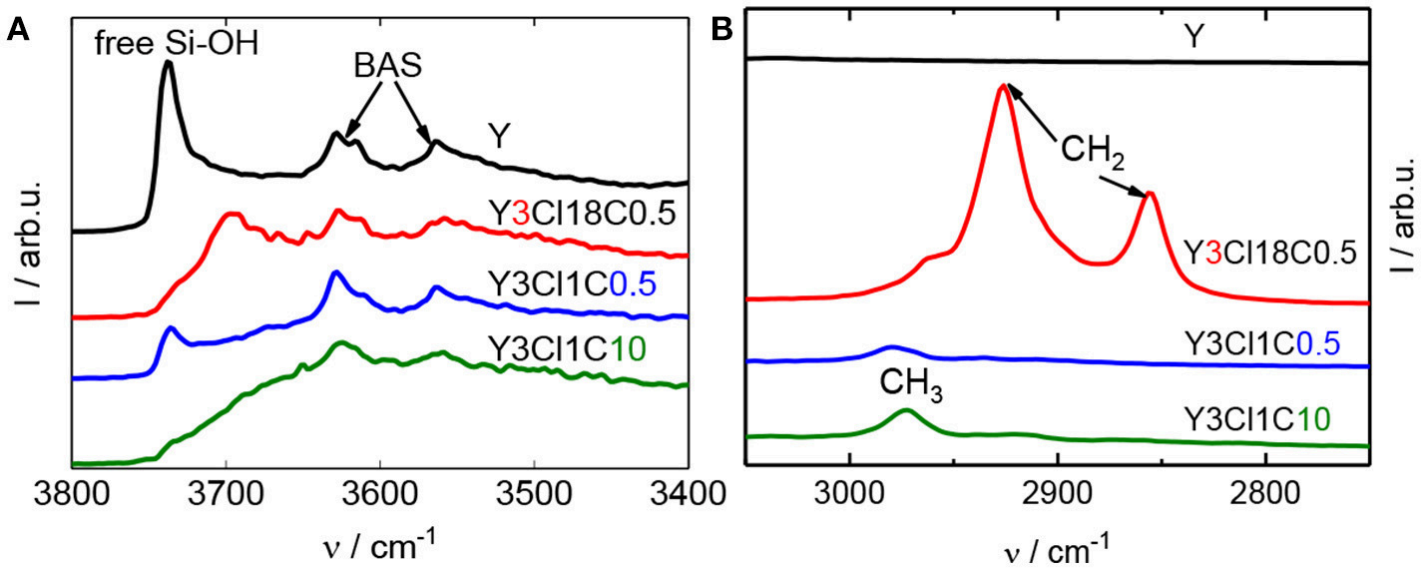
DRIFT spectra at 373 K of zeolite Y and silylated zeolite Y silylated with n-octadecyltrichlorosilane (Y3Cl18C0.5) or methyltrichlorosilane with different silane/zeolite ratios (0.5 or 10 mmol per 1 g of zeolite; Y3Cl1C0.5 and Y3Cl1C10, respectively), showing the O–H stretching **(A)** and C–H stretching bands **(B)**.

The silylation of zeolite Y was conducted using different organosilanes including mono-, di-, trichlorosilanes as well as with different aimed silanes loadings. Figure 1 shows DRIFT spectra for zeolite Y and selected silylated samples with different alkyl chain lengths, e.g., Y3Cl1C0.5 and Y3Cl18C0.5. In the O–H stretching vibration region (Figure 1A), the parent zeolite Y displayed a dominant band at 3,738 cm^−1^, which can be assigned to the free silanol groups, and two other bands characteristic of Brønsted acid sites (BAS) located in the supercages (3,627 cm^−1^) and the sodalite cages (3,562 cm^−1^) of the zeolite framework (Weitkamp, 2000). The band for free silanol groups was present with significantly lower intensity for silylated zeolites using monochlorosilanes (cf. Figure S.1). In these cases, the silylation is found to be selective toward the free surface silanol groups. Interestingly, this band at 3,738 cm^−1^ is remained at the same frequency for Y3Cl1C0.5 with a lower intensity. However, it is slightly shifted to 3,700 cm^−1^ for the silylated zeolite using n-octadecyltrichlorosilane (Figure 1A). This shift of the band indicates the presence of unreacted silanol groups due to the fact that this bulky silylating agent might experience difficulty to reach all free silanol groups. The shift in the frequency was claimed to result from the interaction between the unreacted Si–OH and the attached alkylsilyl groups (Zapata et al., 2012). Changing the amount of methyltrichlorosilane applied in the silylation from 0.5 to 10 mmol g^−1^, i.e., Y3Cl1C10, the band representative of the free silanol groups was no longer found. Therefore, it can be assumed that all accessible Si-OH groups were grafted. The two above-mentioned O–H vibration bands associated with BAS remain present for all silylated samples, which indicates that these internal sites remained unaffected during the silylation. In coherence with the results of Zapata et al. (2012), characteristic bands were found for the silylated zeolites in the C–H vibration range, as depicted in Figure 1B. The band centered at 2,974 cm^−1^ is corresponding to the C–H stretching vibration of methyl (CH_3_) groups and is shifted to 2,964 cm^−1^ for Y3Cl18C0.5. For silanes with long alkyl chains this spectral region is dominated by bands at 2,928 and 2,856 cm^−1^ assigned to the methylene (CH_2_) groups. As expected, all these bands are absent in the spectrum of the parent zeolite. These observations in the O–H and C–H vibration range for silylated zeolites can be taken as an indication of the attachment of the alkylsilyl groups to the silanol groups of the external zeolite surface (Zapata et al., 2012).

**Figure 2:**
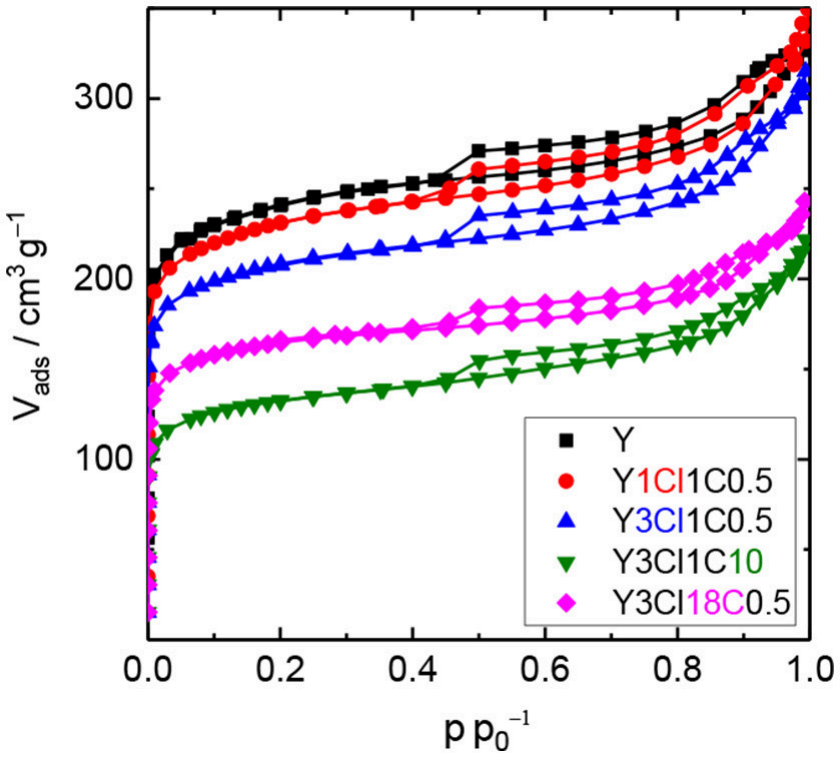
N_2_ physisorption isotherms of zeolite Y and zeolite Y silylated with trimethylmonochlorosilane (Y1Cl1C0.5), methyltrichlorosilane with different silane/zeolite ratios (0.5 or 10 mmol per 1 g of zeolite; Y3Cl1C0.5 and Y3Cl1C10, respectively) or n-octadecyltrichlorosilane (Y3Cl18C0.5).

N_2_ physisorption experiments were conducted with the silylated materials to investigate the effect of the introduced organosilanes on the textural properties. Figure 2 shows the N_2_ physisorption isotherms for the parent zeolite Y and four silylated samples. Similar to zeolite Y, silylated samples exhibit combined type I and type II isotherms with a steep rise in the adsorbed N_2_ volume at p ${\text{p}}_{0}^{-1}$ < 0.01, an uptake at a relative pressure above 0.7 as well as a type H4 hysteresis loop. Silylation with monochlorosilanes especially does not have a strong influence on the textural properties of the zeolite (cf. Table 1). The specific volume of the mesopores was found virtually unaffected remaining ~0.23 cm^3^ g^−1^. On the other hand, the specific micropore volume was reduced from 0.28 to 0.20 cm^3^ g^−1^ for Y1Cl18C0.5. The loss of microporosity is also correlated to the loss of the specific surface area to a large extent. The highest decrease in the specific surface area of up to 25% was found for Y1Cl18C0.5. This might be explained by pore blockage as a result of organosilane introduction.

**Table 1:** Textural properties, i.e., specific surface area (A_BET_), specific micropore surface area (A_micro_), total specific pore volume (V_total_), specific micropore volume (V_micro_), specific mesopore volume (V_meso_), and difference in A_BET_, V_total_ compared to parent zeolite Y (ΔA_BET_, Δ V_total_) of zeolite Y before and after silylation.

**Sample**	**A_BET_^a^ /m^2^ g^−1^**	**ΔA_BET_^a^ /%**	**A_micro_^b^ /m^2^ g^−1^**	**V_total_^c^ /cm^3^ g^−1^**	**Δ V_total_^c^ /%**	**V_micro_^b^ /cm^3^ g^−1^**	**V_meso_^d^ /cm^3^ g^−1^**
Y	788	0	574	0.51	0	0.28	0.23
Y1Cl1C0.5	715	9	506	0.49	4	0.27	0.22
Y1Cl1C1.5	725	8	430	0.47	8	0.22	0.25
Y1Cl3C0.5	633	20	431	0.47	8	0.23	0.24
Y1Cl8C0.5	565	28	387	0.44	14	0.20	0.24
Y1Cl18C0.5	564	28	415	0.38	25	0.20	0.18
Y1Cl18C1	589	25	430	0.40	22	0.21	0.19
Y2Cl18C0.5	436	45	297	0.35	31	0.16	0.19
Y3Cl1C0.5	644	18	450	0.49	4	0.24	0.25
Y3Cl1C10	411	48	285	0.34	33	0.15	0.19
Y3Cl18C0.5	507	36	359	0.35	31	0.19	0.16

a *via BET*.

b *via t-plot*.

c *via Single point*.

d *V_meso_ = V_total_ − V_micro_*.

The decrease in the microporosity after silylation is more intense when changing from mono- to the corresponding trichlorosilanes (cf. Figure S.2). Significant losses of 37% in the specific micropore surface area and 32% in the specific micropore volume were evident for Y3Cl18C0.5. In addition, the mesoporosity was also affected, which contributed to the loss of 36% in the total specific surface area and 31% in the total specific pore volume of this material. Most probably the drastic loss in both micro- and mesopore volume can be explained by the formation of a polymeric layer due to the condensation of trichlorosilanes during silylation making the pores inaccessible for N_2_ (Yoshida et al., 2001). Noticeably, this effect is most pronounced for the sample Y3Cl1C10, silylated with an excess amount of (CH_3_)SiCl_3_.

**Figure 3:**
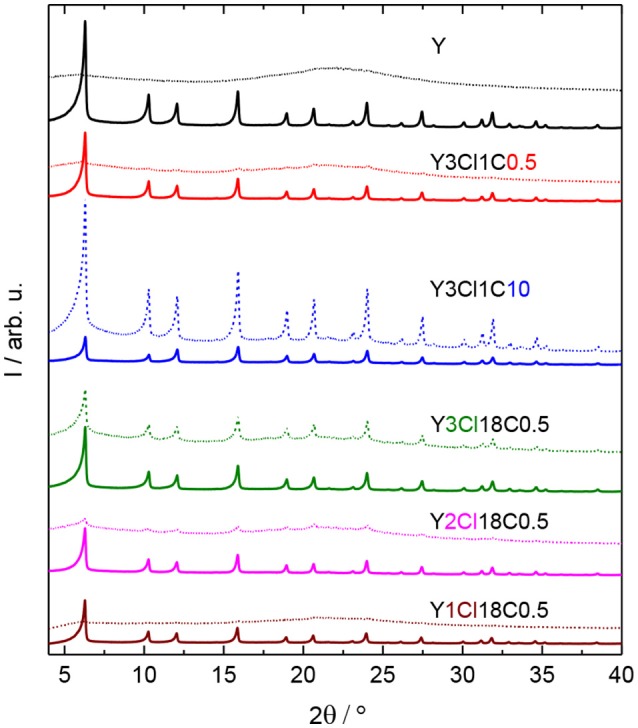
XRD patterns of zeolite Y and zeolite Y silylated with n-octadecyltrichlorosilane (Y3Cl18C0.5) or methyltrichlorosilane with different silane/zeolite ratios (0.5 or 10 mmol per 1 g of zeolite; Y3Cl1C0.5 and Y3Cl1C10, respectively) before (solid lines) and after (dashed lines) the stability test in the aqueous solution of 0.2 M LA and 0.6 M FA at 473 K, autogenous pressure for 24 h.

In addition, XRD patterns of silylated zeolites showed major reflections at 2θ = 6.3, 10.3, and 15.9° corresponding to the (111), (220), and (331) lattice planes of the faujasite framework topology. This observation indicated no considerable change in structural property of the zeolites after silylation (cf. Figure 3).

### Hydrothermal stability of silylated zeolite Y in an acidic aqueous solution

In the stability test, zeolite Y and silylated zeolite Y were exposed to the reactant solution containing 0.2 M LA and 0.6 M FA at 473 K for 24 h. After the stability test, the characteristic reflections for Y-type zeolite framework can no longer be found in the XRD patterns for both zeolite Y and zeolite Y silylated with monochlorosilanes, e.g., Y1Cl18C0.5 (cf. Figure 3, Figure S.4). A broad background typical for amorphous material is visible, which indicates the destruction of the crystalline zeolite framework. By increasing the number of Cl atom of the organosilanes applied in the silylation, the materials, e.g., Y2Cl18C0.5 and Y3Cl18C0.5, obtained after the stability test still exhibited the characteristic reflections of the faujasite framework topology. However the characteristic reflections possess a lower intensity emerging from a broad background. This indicates a partial retention of the zeolite framework. Y3Cl18C0.5 appeared to retain the crystalline zeolite structure to a much higher degree after the stability test than the parent zeolite Y. However, the broad underlying background representative of the amorphous, non-microporous solid typically formed via framework destruction is still evident. In contrast, Y3Cl1C10 showed the highest relative degree of crystallinity among the samples, indicating that the high-loading silylation of zeolite Y with (CH_3_)SiCl_3_ seems to successfully result in a comparatively stable material.

**Figure 4:**
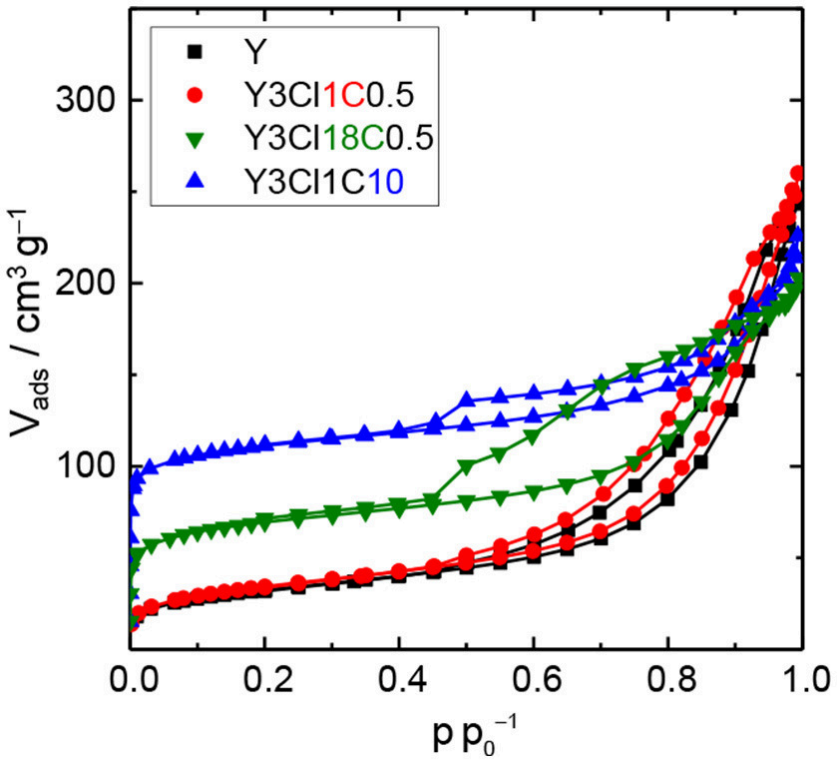
N_2_ physisorption isotherms of zeolite Y and zeolite Y silylated with n-octadecyltrichlorosilane (Y3Cl18C0.5) or methyltrichlorosilane with different silane/zeolite ratios (0.5 or 10 mmol per 1 g of zeolite; Y3Cl1C0.5 and Y3Cl1C10, respectively) after the stability test in the aqueous solution of 0.2 M LA and 0.6 M FA at 473 K, autogenous pressure for 24 h.

The observations from XRD are in agreement with the N_2_ physisorption data shown in Figure 4. From the isotherm of the zeolite Y after the stability test, the absence of the steep increase in adsorbed N_2_ volume at p ${\text{p}}_{0}^{-1}$ < 0.01 is indicative of the total loss of microporosity. In addition, the observation of a H3 type hysteresis loop might be indicate the formation of macropores in the spent zeolite Y (Thommes et al., 2015). Accordingly, significant losses of about 86% in the total specific surface area and 31% in total specific pore volume were found. Therefore, it can be concluded that the parent zeolite Y is hydrothermally unstable undergoing complete framework destruction when exposed to the acidic aqueous conditions in the stability test. However, the silylation has a positive effect on the hydrothermal stability of zeolite Y. The N_2_ physisorption isotherm for Y3Cl18C0.5 after the stability test displayed a lower uptake of adsorbed nitrogen at p ${\text{p}}_{0}^{-1}$ < 0.01 and a larger hysteresis loop indicative of larger mesopores compared to the fresh counterpart (cf. Figure 2). Additionally, application of t-plot model evidently confirmed a decrease in the specific micropore surface area by 251 m^2^ g^−1^ and a slight increase in the specific mesopore volume by 0.07 cm^3^ g^−1^ compared to the fresh Y3Cl18C0.5 (Table 2). The deterioration in the textural structure of Y3Cl18C0.5 after the stability test is probably caused by the partial hydrothermal deconstruction of the zeolite framework to a large extent. By silylation using trichloromethylsilane (Y3Cl1C0.5) a complete loss of 0.24 cm^3^ g^−1^ in microporosity could not be prevented. As confirmed by XRD (cf. Figure 3) this loss can be mainly attributed to framework destruction. However, silylation using 3Cl1C with an excess amount, i.e., 10 mmol per 1 g of zeolite (Y3Cl1C10) resulted in an almost complete retention of the zeolite structure after the stability test as indicated by the obtained similarity in the shape of the corresponding N_2_ physisorption isotherm when compared to the fresh sample. Interestingly, the specific mesopore volume was found to be the same (0.19 cm^3^ g^−1^) for Y3Cl1C10 before and after stability test with a slight decrease of 0.03 cm^3^ g^−1^ in the specific micropore volume. In comparison, for Y3Cl18C0.5 the corresponding loss was more pronounced with 0.13 cm^3^ g^−1^ which is in coherence with the observation from XRD pattern proving that Y3Cl1C10 is more stable than Y3Cl18C0.5. Most probably the more pronounced framework destruction is due to the availability of the unreacted silanol groups in Y3Cl18C0.5 caused by the bulky shape of the n-octadecylsilyl groups inhibiting complete silylation of silanol groups as mentioned before in section Impact of Attached Organosilanes on Textural and Structural Properties of Zeolite Y. Among the silylated zeolites, Y3Cl18C0.5 and Y3Cl1C10 are the most stable materials under these APP related conditions. Hence, to investigate in the impact of the silylation on the catalytic activity, these two silylating agents were applied over 3wt.-% Pt supported on zeolite Y.

**Table 2:** Textural properties, i.e., specific surface area (A_BET_), specific micropore surface area (A_micro_), total specific pore volume (V_total_), specific micropore volume (V_micro_), specific mesopore volume (V_meso_), and difference in A_BET_, V_total_ compared to fresh counterpart (ΔA_BET_, Δ V_total_), of zeolite Y and silylated zeolite Y after the stability test at 473 K and autogenous pressure for 24 h.

**Sample**	**A_BET_^a^ /m^2^ g^−1^**	**ΔA_BET_^a^ /%**	**A_micro_^b^ /m^2^ g^−1^**	**V_total_^c^ /cm^3^ g^−1^**	**Δ V_total_^c^ /%**	**V_micro_^b^ /cm^3^ g^−1^**	**V_meso_^d^ /cm^3^ g^−1^**
Y	113	86	0	0.35	31	0	0.35
Y1Cl1C1.5	143	80	0	0.38	19	0	0.38
Y1Cl18C1	87	85	0	0.32	20	0	0.32
Y2Cl18C0.5	144	67	0	0.28	20	0	0.28
Y3Cl1C0.5	120	81	0	0.35	29	0	0.35
Y3Cl1C10	346	16	235	0.31	9	0.12	0.19
Y3Cl18C0.5	223	56	108	0.29	17	0.06	0.23

a *via BET*.

b *via t-plot*.

c *via Single point*.

d *V_meso_ = V_total_ – V_micro_*.

### Catalytic activity of silylated Pt on zeolite Y catalysts in the hydrogenation of LA

**Figure 5:**
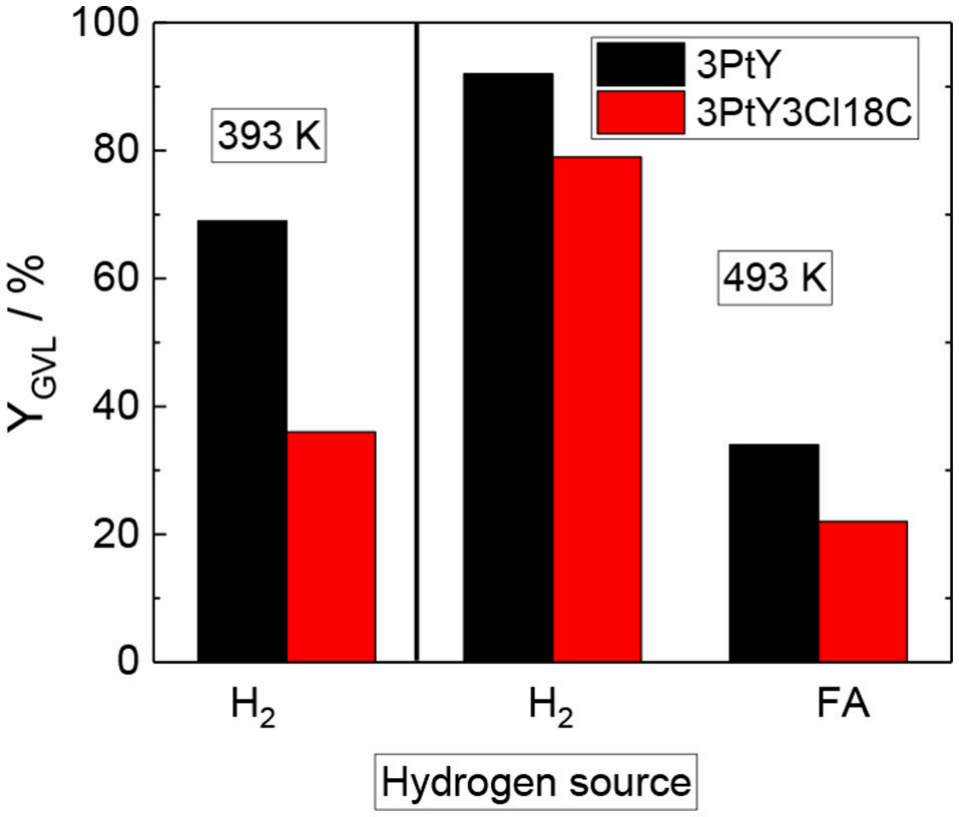
GVL yield (Y_GVL_) over 3PtY and 3PtY3Cl18C0.5, using different reducing agents i.e., H_2_ (2.5 MPa) or FA (0.6 M) (reaction conditions: V_solution_ = 125 cm^3^, cLA = 0.2 M, T = 393 K, or 493 K, m_catalyst_ = 0.5 g, n = 700 min^−1^, 24 h).

To study the impact of silylation on the catalytic activity of silylated catalysts, 3PtY and 3PtY3Cl18C0.5, 3PtY3Cl1C10 were used in the aqueous-phase hydrogenation of LA. The LA conversion, the GVL yield and the selectivity toward GVL after 24 h are displayed in Table 3 and Figure 5. Notably, no other product besides GVL was found in the aqueous product solution after the catalytic experiments as confirmed by the HPLC, ^1^H NMR, and ^13^C NMR (cf. Figures S.7, S.8). However, non-soluble carbonaceous deposits were obtained after hydrogenation of LA over all catalysts tested. These non-soluble carbonaceous deposits, presumably humins, are most likely the side products leading to the difference between LA conversion and GVL yield.

**Table 3:** LA conversion (X_LA_), GVL yield (Y_GVL_), and selectivity toward GVL (S_GVL_) in the hydrogenation of LA using different reducing agents, i.e., FA or H_2_, over 3PtY and 3PtY silylated with n-octadecyltrichlorosilane (3PtY3Cl18C0.5) or methyltrichlorosilane with different silane/zeolite ratios (0.5 or 10 mmol per 1 g of 3PtY; 3PtY3Cl1C0.5 and 3PtY3Cl1C10, respectively).

**Catalysts**	Pt loading^*^ /wt.-%	**Reducing agent**	**T /K**	**X_LA_ /%**	**Y_GVL_ /%**	**S_GVL_ /%**
Y	n.d.	FA	493	n.d.	13	n.d.
3PtY	2.7	FA	493	42	34	80
		H_2_	493	100	92	92
		H_2_	393	95	69	73
3PtY3Cl18C0.5	2.5	FA	493	42	22	52
		H_2_	493	97	79	81
		H_2_	393	60	36	60
3PtY3Cl1C10	2.4	FA	493	38	12	32

*Reaction conditions: reducing agent H_2_ (2.5 MPa) or FA (0.6 M), V_solution_ = 125 cm^3^, c_LA_ = 0.2 M, m_catalyst_ = 0.5 g, n = 700 min^−1^, 24 h, n.d., not determined*,

* *via ICP-OES*.

The hydrogenation of LA over 3PtY was conducted using different reducing agents, i.e., FA and H_2_. In the presence of FA, the GVL yield was found to be much lower compared to that obtained when H_2_ was used as the reducing agent, i.e., 34 vs. 92%, respectively. The significantly decreased GVL yield in hydrogenation of LA using FA as the reducing agent was also reported over Pd, Pt, Ru based catalysts on carbon by Ruppert et al. (2015). They suggested that CO formed during the decomposition of FA poisons the Pt catalyst (Du et al., 2011). Another important factor might be the competitive adsorption of FA and LA on the active sites of the catalyst surface. In comparison to unsilylated catalysts, the silylated counterparts exhibited lower catalytic performance, e.g., a GVL yield of 22% was observed over 3PtY3Cl18C0.5 after 24 h of reaction at 493 K using FA as a reducing agent. Under the same reaction conditions, 12% of GVL yield could be achieved over 3PtY3Cl1C10. This drastically lower activity of silylated catalysts is caused by a combination of silylation-induced changes in the catalyst's properties. Firstly, 3PtY3Cl1C10 displayed a much lower specific surface area (483 m^2^ g^−1^) compared to 3PtY (775 m^2^ g^−1^) due to the blockage of micropores as described above (cf. section Impact of Attached Organosilanes on Textural and Structural Properties of Zeolite Y). Secondly, the attachment of organosilanes on the surface might also reduce the accessibility of active Pt sites. Thirdly, after silylation a decrease in the Pt loading of 16%, probably due to the additionally attached organosilanes, was obtained via ICP-OES for 3PtY3Cl18C0.5 in comparison to 3PtY. This decrease in the Pt loading might partly explain the drastically lower hydrogenation activity. Furthermore, silylation changes the wettability of the materials. The apparently hydrophobic catalysts obtained after silylation, therefore, impose a hydrophobic barrier which may negatively influence the catalytic activity.

The presence of FA was found to considerably impede the aqueous-phase hydrogenation of LA. Therefore, in later catalytic experiments H_2_ was used as the reducing agent in the absence of FA under similar reaction conditions, i.e., 125 cm^3^ of LA 0.2 M, at 493 K with 2.5 MPa H_2_ for 24 h (cf. Table 3 and Figure 5). In contrast to the LA conversion achieved using FA as reducing agent, after 24 h over both 3PtY as well as 3PtY3Cl18C0.5 an almost complete LA conversion is observed when using H_2_. However, over the silylated catalyst 3PtY3Cl18C0.5 a lower GVL yield of 79% was observed compared to 92% of GVL yield obtained over 3PtY. Interestingly, if the hydrogenation using H_2_ is conducted at 393 K, over 3PtY the LA conversion remains almost unchanged at a considerably lower GVL yield of 69%. Over 3PtY3Cl18C0.5 at 393 K both the LA conversion as well as GVL yield are largely impact amounting to 60 and 36%, respectively.

**Figure 6:**
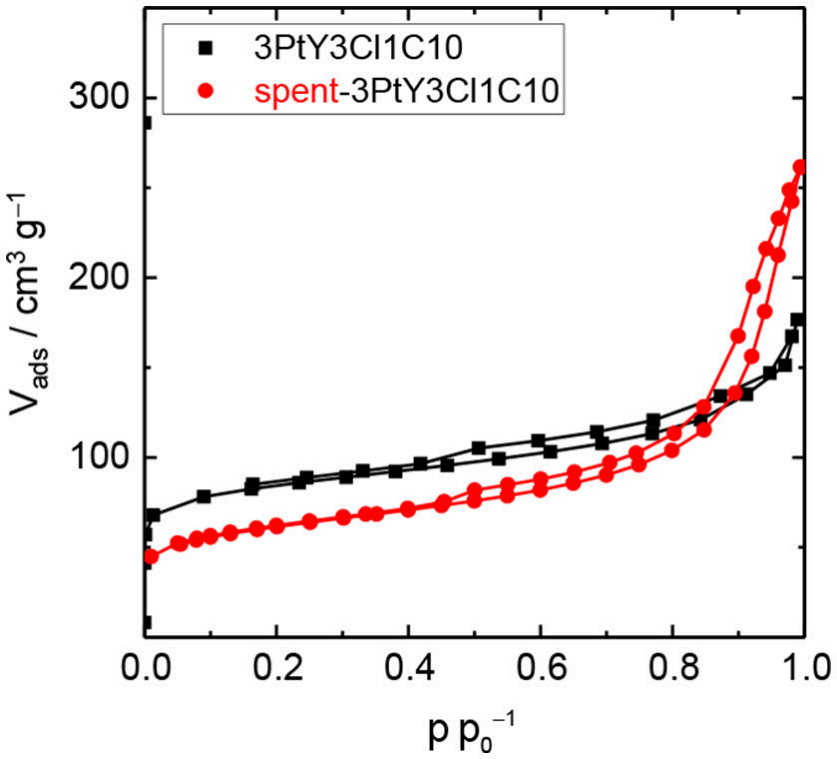
N_2_ physisorption isotherms of 3PtY3Cl1C10 before and after the hydrogenation of LA (reaction conditions: V_solution_ = 125 cm^3^, c_LA_ = 0.2 M, c_FA_ = 0.6 M, T = 493 K, m_catalyst_ = 0.5 g, *n* = 700 min^−1^, 24 h).

To investigate the hydrothermal stability of these catalysts after catalytic experiments, spent catalysts were collected and characterized via N_2_ physisorption (cf. Figure 6, Figure S.5) and XRD (cf. Figure S.5). N_2_ physisorption results revealed that the silylated catalyst using 3Cl18C as silylating agent was not stable at 493 K, under autogenous pressure after 24 h in the presence of FA. Similar to 3PtY, a decrease of 91% in the total specific surface area (44 vs. 483 m^2^ g^−1^) and 70% in total specific pore volume (0.11 vs. 0.37 cm^3^ g^−1^) were found for 3PtY3Cl18C0.5 (cf. Table S.2). The severe degradation of the faujasite framework is further confirmed by the absence of the characteristic reflections in the XRD patterns of these samples (cf. Figure S.5). When using only H_2_ as the reducing agent for the hydrogenation of LA at the same reaction temperature of 493 K or even in the case of lower temperature, i.e., 393 K, the absence of the characteristic reflections in the XRD indicative for the faujasite framework topology is also observed for 3PtY3Cl18C0.5 (cf. Figure S.5). Moreover, the total specific surface area decreased from 483 to 91 m^2^ g^−1^ at 493 K and to 76 m^2^ g^−1^ at 393 K, which is consistent with the loss in the total specific pore volume from 0.37 to 0.25 cm^3^ g^−1^ at 493 K and to 0.31 cm^3^ g^−1^ at 393 K as a result of the structural collapse as confirmed by XRD. On the other hand, as can be seen in the N_2_ isotherm for 3PtY3Cl1C10 a plateau was observed after a slight increase in the adsorbed N_2_ volume at p ${\text{p}}_{0}^{-1}$ < 0.01, which is indicative of a partial retention of microporosity compared to 3PtY3Cl18C0.5 (Figure 6). Application of t-plot model evidenced the preservation of 50% of specific micropore volume for 3PtY3Cl1C10 after hydrogenation of LA in the presence of FA at 493 K under autogenous pressure for 24 h. In spite of the presence of the characteristic reflections of faujasite framework in the XRD pattern for this material, the shape of the baseline indicates that the crystalline zeolite phase was partly converted into an amorphous solid. However, the silylation using 3Cl1C with an excess amount could considerably enhance the hydrothermal stability of zeolite Y compared to 3PtY3Cl18C0.5.

## Conclusions

We demonstrated that selective silylation of the free silanol groups on the external surface is evident by DRIFTS for zeolite Y silylated with monochlorosilanes. Nonetheless, zeolite Y silylated with monochlorosilanes were still prone to the degradation within 24 h under APP related conditions in the stability test, i.e., in an aqueous solution of 0.2 M LA and 0.6 M FA at 473 K under autogenous pressure. In contrast, by silylation with trichlorosilanes the hydrothermal stability of zeolite Y can be improved significantly. For zeolite Y functionalized with n-octadecyltrichlorosilane the destruction of the zeolite structure was found to be considerably retarded probably due to the partial polymerization of the attached organosilanes, which is feasible with more than one chlorine atom per silane. Noticeably, zeolite Y modified with methyltrichlorosilane in an excess amount, e.g., 10 mmol per gram of zeolite, was found to be the most stable material under APP conditions in the stability test.

In the hydrogenation of LA to GVL over 3 wt.-% Pt on zeolite Y silylated either with n-octadecyltrichlorosilane or methyltrichlorosilane using different reducing agents, e.g., FA or H_2_, the stabilizing effect of the silylation is far less pronounced. Only by applying an excess amount of methyltrichlorosilane, i.e., 10 mmol per 1 g of zeolite (3PtY3Cl1C10), a recognizable improvement of the stability of the 3PtY catalyst could be achieved in the LA hydrogenation using FA as reducing agent at 493 K. However, at a comparable LA conversion of 38 and 42%, the GVL yield observed over 3PtY3Cl1C10 is much lower than for 3PtY, i.e., 12 vs. 34% after 24 h. Since no further products could be evidenced by HPLC, ^1^H as well as ^13^C NMR in the liquid product solution after 24 h of reaction it is most likely that insoluble humins are formed leading to the discrepancy in conversion and yield. In addition, 3PtY3Cl1C10 still suffers from partial damage of the zeolite framework.

These results show, that silylation of zeolite Y using an excess amount of methyltrichlorosilane can improve its hydrothermal stability in aqueous acidic solutions, and, moreover, that 3 wt.-% Pt on zeolite Y silylated in this manner is active in the *in-situ* hydrogenation of LA using FA as reducing agent.

## Author contributions

All authors listed have made a substantial, direct and intellectual contribution to the work, and approved it for publication.

### Conflict of interest statement

The authors declare that the research was conducted in the absence of any commercial or financial relationships that could be construed as a potential conflict of interest.

## References

[B1] Abdelrahman O. A. Heyden A. Bond J. Q. (2014). Analysis of kinetics and reaction pathways in the aqueous-phase hydrogenation of levulinic acid to form γ-valerolactone over Ru/C. ACS Catal. 4, 1171–1181. 10.1021/cs401177p

[B2] Abdelrahman O. A. Luo H. Y. Heyden A. Román-Leshkov Y. Bond J. Q. (2015). Towards rational design of stable, supported metal catalysts for aqueous phase processing: insights from the hydrogenation of levulinic acid. J. Catal. 329, 10–21. 10.1016/j.jcat.2015.04.026

[B3] Alonso D. M. Bond J. Q. Dumesic J. A. (2010). Catalytic conversion of biomass to biofuels. Green Chem. 12, 1493–1513. 10.1039/c004654j

[B4] Alonso D. M. Wettstein S. G. Dumesic J. A. (2013). Gamma-valerolactone, a sustainable platform molecule derived from lignocellulosic biomass. Green Chem. 15, 584–595. 10.1039/c3gc37065h

[B5] Chheda J. N. Huber G. W. Dumesic J. A. (2007). Liquid-phase catalytic processing of biomass-derived oxygenated hydrocarbons to fuels and chemicals. Angew. Chem. Int. Ed. Engl. 46, 7164–7183. 10.1002/anie.200604274 17659519

[B6] Cortright R. D. Davda R. R. Dumesic J. A. (2002). Hydrogen from catalytic reforming of biomass-derived hydrocarbons in liquid water. Nature 418, 964–967. 10.1038/nature01009 12198544

[B7] Delhomme C. Schaper L.-A. Zhang-Preße M. Raudaschl-Sieber G. Weuster-Botz D. Kühn F. E. (2013). Catalytic hydrogenation of levulinic acid in aqueous phase. J. Organomet. Chem. 724, 297–299. 10.1016/j.jorganchem.2012.10.030

[B8] Deng L. Li J. Lai D.-M. Fu Y. Guo Q.-X. (2009). Catalytic conversion of biomass-derived carbohydrates into gamma-valerolactone without using an external H2 supply. Angew. Chem. Int. Ed. Engl. 48, 6529–6532. 10.1002/anie.200902281 19630045

[B9] Deng L. Zhao Y. Li J. Fu Y. Liao B. Guo Q.-X. (2010). Conversion of levulinic acid and formic acid into γ-Valerolactone over heterogeneous catalysts. ChemSusChem 3, 1172–1175. 10.1002/cssc.201000163 20872402

[B10] Du X.-L. He L. Zhao S. Liu Y.-M. Cao Y. He H.-Y.. (2011). Hydrogen-independent reductive transformation of carbohydrate biomass into γ-valerolactone and pyrrolidone derivatives with supported gold catalysts. Angew Chem. Int. Ed. Engl. 50, 7815–7819. 10.1002/anie.201100102 21732502

[B11] Ennaert T. Geboers J. Gobechiya E. Courtin C. M. Kurttepeli M. (2015). Conceptual frame rationalizing the self-stabilization of H-USY zeolites in hot liquid water. ACS Catal. 5, 754–768. 10.1021/cs501559s

[B12] Ennaert T. Van Aelst J. Dijkmans J. de Clercq R. Schutyser W. Dusselier M.. (2016). Potential and challenges of zeolite chemistry in the catalytic conversion of biomass. Chem. Soc. Rev. 45, 584–611. 10.1039/C5CS00859J 26691750

[B13] Galadima A. Muraza O. (2017). Stability improvement of zeolite catalysts under hydrothermal conditions for their potential applications in biomass valorization and crude oil upgrading. Microporous Mesoporous Mater. 249, 42–54.

[B14] Gallezot P. (2012). Conversion of biomass to selected chemical products. Chem. Soc. Rev. 41, 1538–1558. 10.1039/C1CS15147A 21909591

[B15] Geilen F. M. A. Engendahl B. Harwardt A. Marquardt W. Klankermayer J. Leitner W. (2010). Selective and flexible transformation of biomass-derived platform chemicals by a multifunctional catalytic system. Angew. Chem. 122, 5642–5646. 10.1002/ange.201002060 20586088

[B16] Huang B. T. Leveneur S. Zamar T. Mikkola J. P. Taouk B. (2015). Towards Production of γ-valerolactone via hydrogenation of aqueous levulinic acid. Int. J. Chem. React. Eng. 13, 119–127. 10.1515/ijcre-2014-0077

[B17] Huber G. W. Cortright R. D. Dumesic J. A. (2004). Renewable alkanes by aqueous-phase reforming of biomass-derived oxygenates*. Angew. Chem. Int. Ed*. Engl. 43, 1549–1551. 10.1002/anie.200353050 15022230

[B18] Kamm B. Gruber P. R. Kamm M. (eds.) (2005). Biorefineries-Industrial Processes and Products: Status Quo and Future Directions. Weinheim: Wiley-VCH.

[B19] Lange J.-P. Price R. Ayoub P. M. Louis J. Petrus L. Clarke L.. (2010). Valeric biofuels: a platform of cellulosic transportation fuels. Angew Chem. Int. Ed. Engl. 49, 4479–4483. 10.1002/anie.201000655 20446282

[B20] Li W. Xie J.-H. Lin H. Zhou Q.-L. (2012). Highly efficient hydrogenation of biomass-derived levulinic acid to γ-valerolactone catalyzed by iridium pincer complexes. Green Chem. 14, 2388–2390. 10.1039/C2GC35650C

[B21] Luo W. Deka U. Beale A. M. van Eck E. R. Bruijnincx P. C. Weckhuysen B. M. (2013). Ruthenium-catalyzed hydrogenation of levulinic acid: influence of the support and solvent on catalyst selectivity and stability. J. Catal. 301, 175–186. 10.1016/j.jcat.2013.02.003

[B22] Mehdi H. Fábos V. Tuba R. Bodor A. Mika L. T. Horváth I. T. (2008). Integration of homogeneous and heterogeneous catalytic processes for a multi-step conversion of biomass: From sucrose to levulinic acid, γ-valerolactone, 1,4-pentanediol, 2-methyl-tetrahydrofuran, and alkanes. Top Catal. 48, 49–54. 10.1007/s11244-008-9047-6

[B23] Prodinger S. Derewinski M. A. Vjunov A. Burton S. D. Arslan I. Lercher J. A. (2016). Improving stability of zeolites in aqueous phase via selective removal of structural defects. J. Am. Chem. Soc. 138, 4408–4415. 10.1021/jacs.5b12785 26972547

[B24] Ravenelle R. M. Schüβler F. D'Amico A. Danilina N. van Bokhoven J. A. Lercher J. A. (2010). Stability of zeolites in hot liquid water. J. Phys. Chem. C 114, 19582–19595. 10.1021/jp104639e

[B25] Ruppert A. M. Grams J. Jedrzejczyk M. Matras-Michalska J. Keller N. Ostojska K.. (2015). Titania-supported catalysts for levulinic acid hydrogenation: influence of support and its impact on γ-valerolactone yield. ChemSusChem 8, 1538–1547. 10.1002/cssc.201403332 25641864

[B26] Sun Y. Cheng J. (2002). Hydrolysis of lignocellulosic materials for ethanol production: a review. Bioresour. Technol. 83, 1–11. 10.1016/S0960-8524(01)00212-7 12058826

[B27] Thommes M. Kaneko K. Neimark A. V. Olivier J. P. Rodriguez-Reinoso F. Rouquerol J. (2015). Physisorption of gases, with special reference to the evaluation of surface area and pore size distribution (IUPAC Technical Report). Pure Appl. Chem. 87, 1051–1069. 10.1515/pac-2014-1117

[B28] Weitkamp J. (2000). Zeolites and catalysis. Solid State Ionics 131, 175–188. 10.1016/S0167-2738(00)00632-9

[B29] Wright W. R. H. Palkovits R. (2012). Development of heterogeneous catalysts for the conversion of levulinic acid to γ-valerolactone. ChemSusChem 5, 1657–1667. 10.1002/cssc.201200111 22890968

[B30] Yoshida W. Castro R. P. Jou J.-D. Cohen Y. (2001). Multilayer alkoxysilane silylation of oxide surfaces. Langmuir 17, 5882–5888. 10.1021/la001780s

[B31] Zapata P. A. Huang Y. Gonzalez-Borja M. A. Resasco D. E. (2013). Silylated hydrophobic zeolites with enhanced tolerance to hot liquid water. J. Catal. 308, 82–97. 10.1016/j.jcat.2013.05.024

[B32] Zapata P. A. Faria J. Ruiz M. P. Jentoft R. E. Resasco D. E. (2012). Hydrophobic zeolites for biofuel upgrading reactions at the liquid-liquid interface in water/oil emulsions. J. Am. Chem. Soc. 134, 8570–8578. 10.1021/ja3015082 22548687

[B33] Zhang L. Chen K. Chen B. White J. L. Resasco D. E. (2015). Factors that determine zeolite stability in hot liquid water. J. Am. Chem. Soc. 137, 11810–11819. 10.1021/jacs.5b07398 26301890

