# Supplementary material for: Silylated Zeolites With Enhanced Hydrothermal Stability for the Aqueous-Phase Hydrogenation of Levulinic Acid to γ-Valerolactone
Source: Front Chem. 2018 May 16;6:143. doi: 10.3389/fchem.2018.00143 (PMC5964160; doi:10.3389/fchem.2018.00143)
Supplement: Supplementary file 1 [file Data_Sheet_1.pdf]

Supplementary Material

Silylated Zeolites with Enhanced Hydrothermal Stability for the Aqueous-Phase Hydrogenation of Levulinic Acid to γ-Valerolactone

Hue-Tong Vu, Florian Maximilian Harth, Nicole Wilde*

Institute of Chemical Technology, Universität Leipzig, Linnéstraße 3, 04103 Leipzig, Germany

*** Correspondence:** Dr. Nicole Wilde: nicole.wilde@uni-leipzig.de

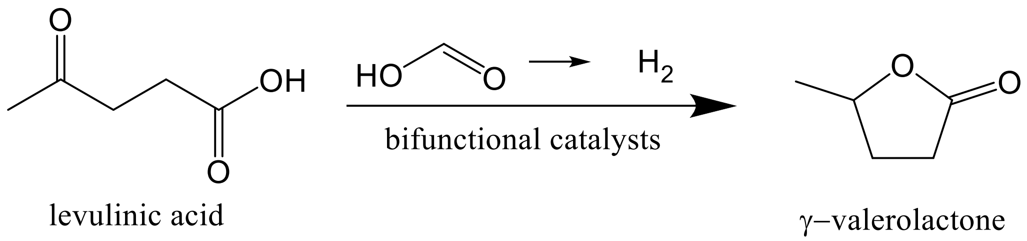

Scheme S.1: Hydrogenation of levulinic acid (LA) towards γ-valerolactone (GVL) using formic acid as a hydrogen source.

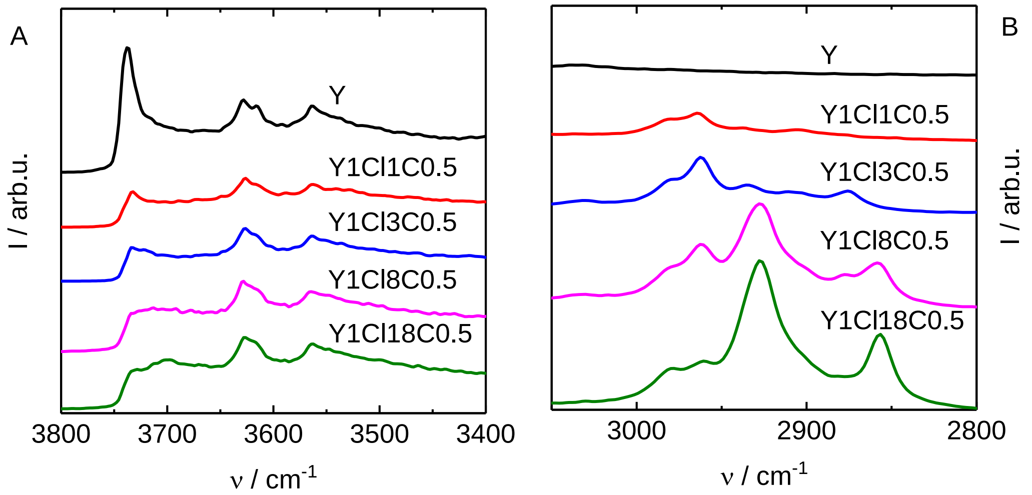

Fig S.1: DRIFT spectra at 373 K of zeolite Y and zeolite Y silylated with chlorotrimethylsilane (Y1Cl1C0.5), chloro(dimethyl)(n-propyl)silane (Y1Cl3C0.5), chloro(dimethyl)(n-octyl)silane (Y1Cl8C0.5) and chlorodimethyl(n-octadecyl)silane (Y1Cl18C0.5), showing the O-H stretching (A) and C-H stretching bands (B).

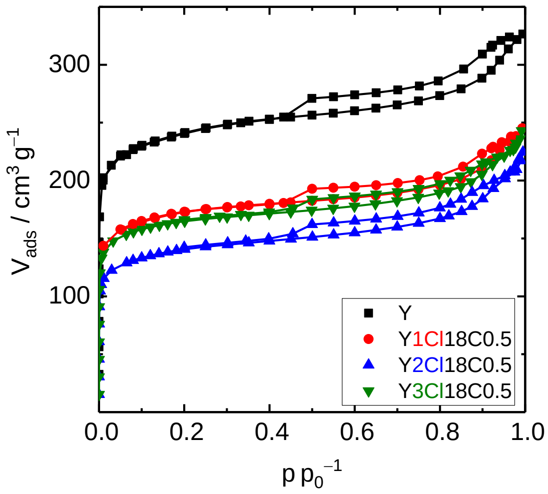

Fig. S.2: N_2 physisorption isotherms of zeolite Y and zeolite Y silylated with chloro-(dimethyl)(n-octadecyl)silane (Y1Cl18C0.5), dichloromethyl(n-octadecyl)silane (Y2Cl18C0.5) and n-octadecyltrichlorosilane (Y3Cl18C0.5).

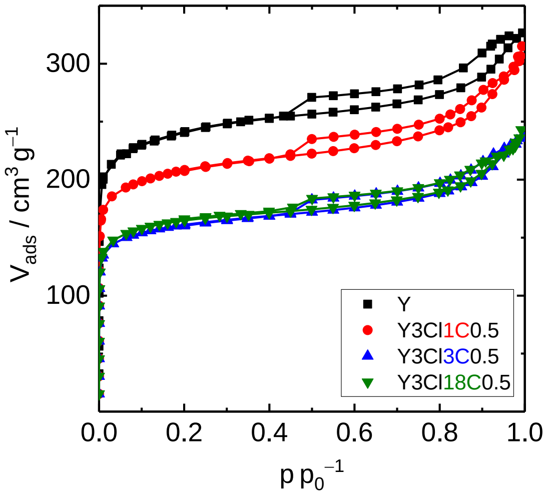

Fig. S.3: N_2 physisorption isotherms of zeolite Y and zeolite Y silylated with methyltrichlorosilane (Y3Cl1C0.5), n-propyltrichlorosilane (Y3Cl3C0.5) and n-octadecyltrichlorosilane (Y3Cl18C0.5).

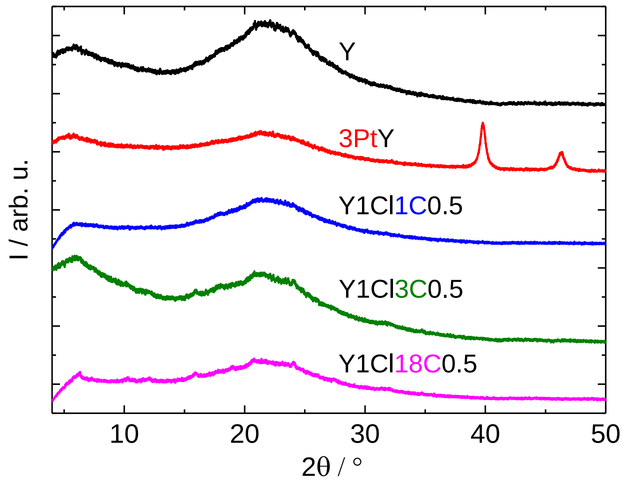

Fig. S.4: XRD patterns of zeolite Y, 3PtY and zeolite Y silylated with chlorotrimethylsilane (Y1Cl1C0.5), chloro(dimethyl)(n-propyl)silane (Y1Cl3C0.5) and chlorodimethyl-(n-octadecyl)silane (Y1Cl18C0.5) after stability tests in the aqueous solution of 0.2 M LA and 0.6 M FA at 473 K, autogenous pressure for 24 h.

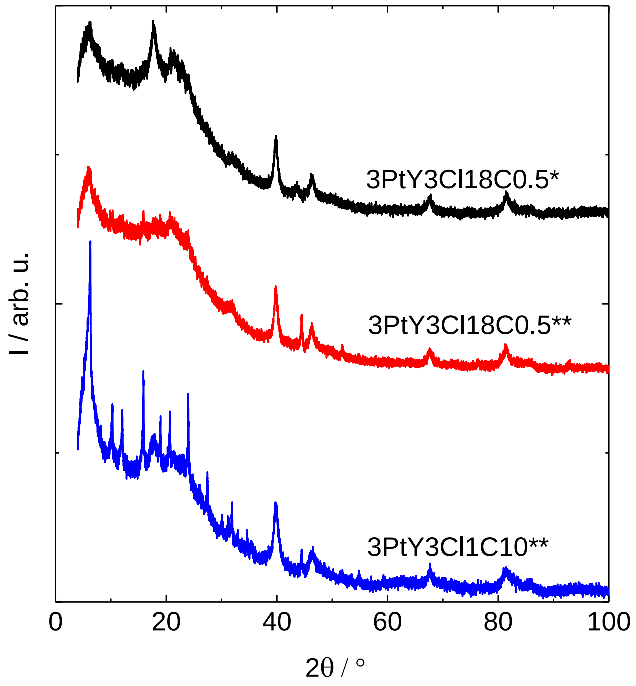

Fig. S.5: XRD patterns of silylated 3Pt/Y after the hydrogenation of LA using different reducing agents, e.g., *H_2 (2.5 MPa) or **FA (0.6 M) (reaction conditions: $V_{solution}$ = 125 cm^3, c_{LA} = 0.2 M, T = 493 K, $m_{catalyst}$ = 0.5 g, n = 700 min^{-1}, 24 h).

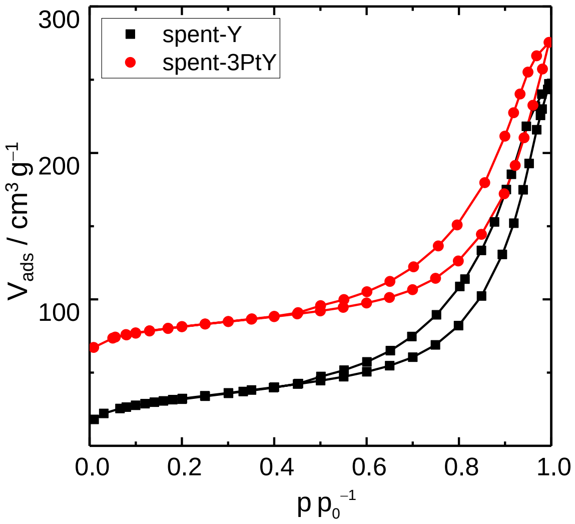

Fig. S.6: N_2 physisorption isotherms of 3PtY after the stability test in the aqueous solution of 0.2 M LA and 0.6 M FA at 473 K, autogenous pressure for 24 h. For clarification, the N_2 physisorption isotherm of 3PtY was shifted upward by 50 $cm^3\ g^{-1}$.

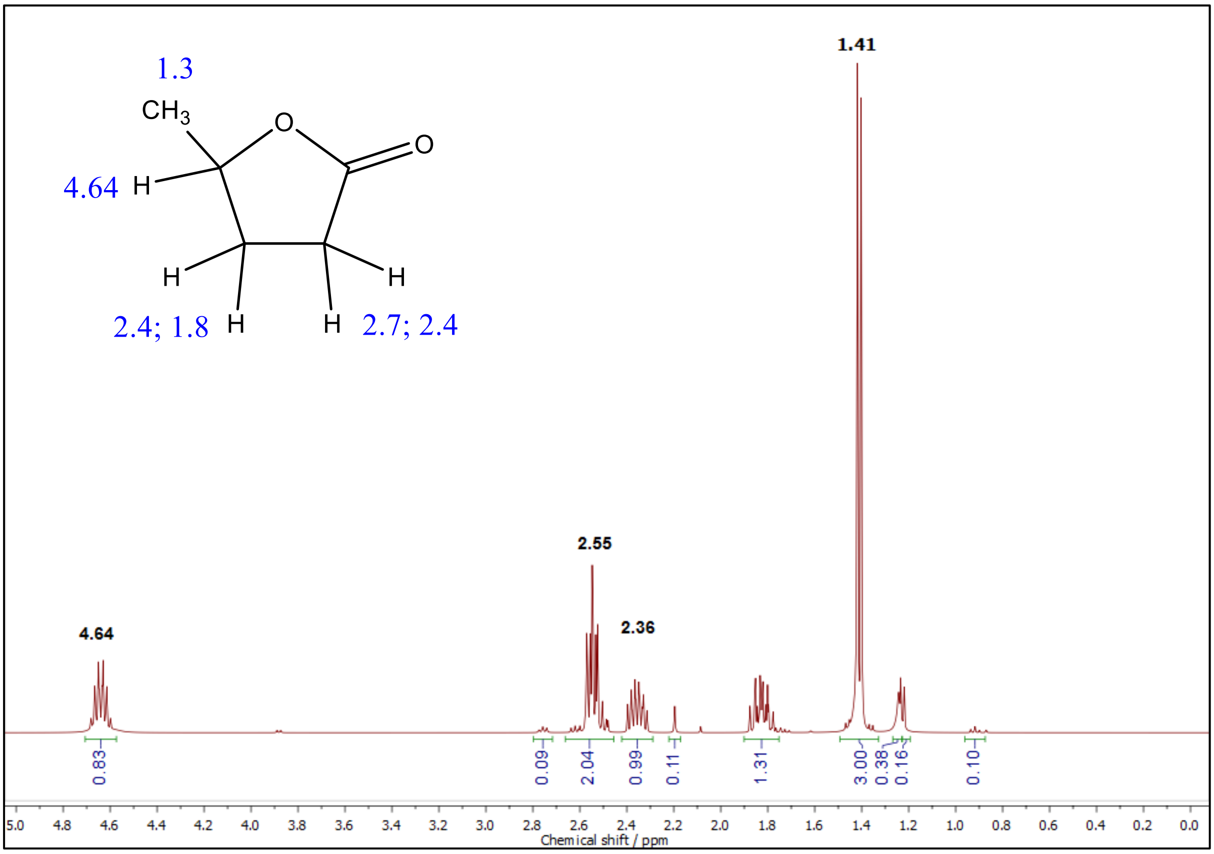

Fig. S.7: ^{1}H NMR spectrum (400 MHz, $CDCl_3$) of the product solution after the hydrogenation of LA using H_2 (2.5 MPa) over 3PtY at 493 K for 24 h: δ 4.6 (m, 1H), δ 2.5 (m, 2H), δ 2.4 (m, 1H), δ 1.4 (d, 3H)

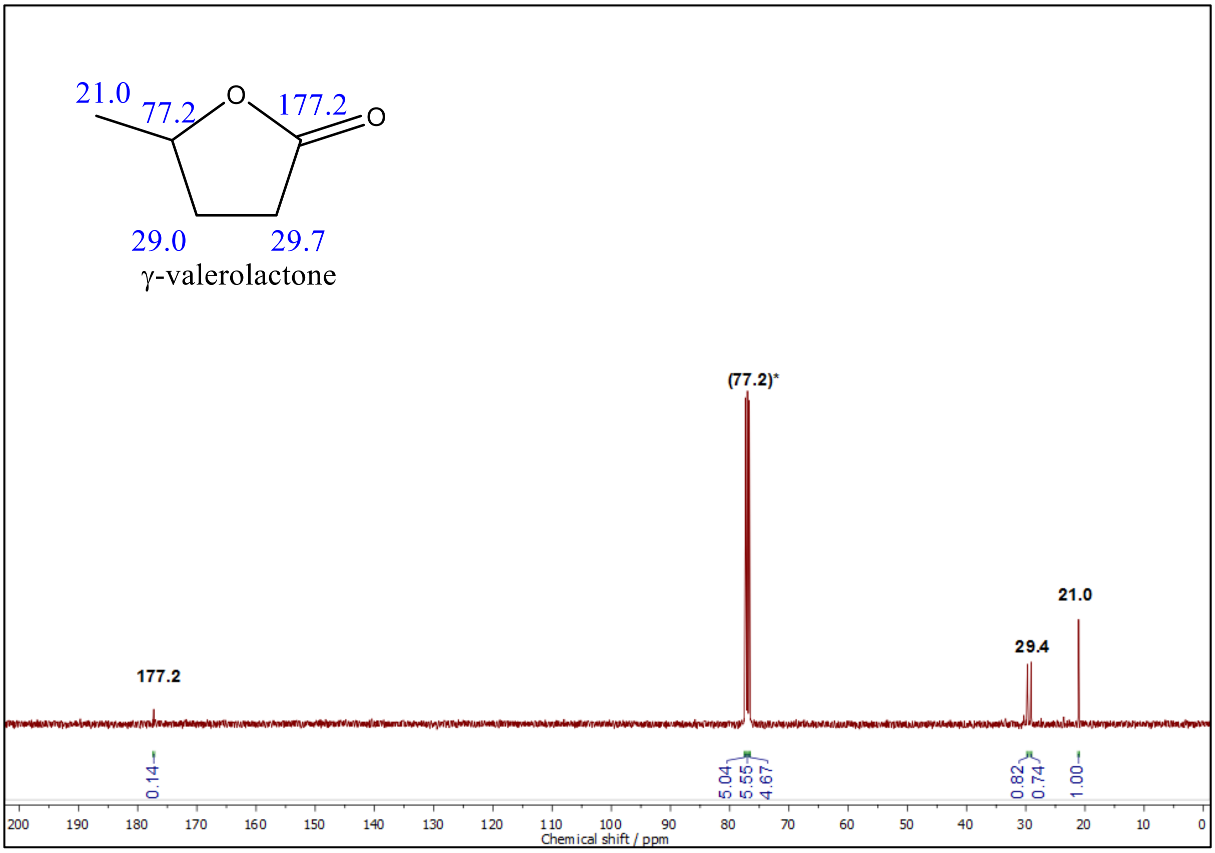

Fig. S.8: ^{13}C NMR spectrum (101 MHz, $CDCl_3$) of the product solution after the hydrogenation of LA using H_2 (2.5 MPa) over 3PtY at 493 K for 24 h: δ 177.2 (s, 1C), δ 77.2 (s, 1C) (overlapped with δ 77.3, δ 77.0, δ 76.7 (s, 1C) of $CDCl_3$), δ 29.7 (s, 1C), δ 29.1 (s, 1C), δ 21.0 (s, 1H)

Tab. S.1: Notation of silylated zeolite Y materials with respective silylating agents and aimed loadings.

Sample	Silylating agents	Supplier	Purity / %	Aimed silaneloading / mmol g^{-1}
Y1Cl1C0.5	$(CH_3)_3SiCl$	TCI	> 90	0.5
Y1Cl1C1.5	$(CH_3)_3SiCl$	TCI		1.5
Y1Cl3C0.5	$(n\text{-}C_3H_7)(CH_3)_2SiCl$	ABCR	97	0.5
Y1Cl8C0.5	$(n\text{-}C_8H_{17})(CH_3)_2SiCl$	Aldrich	95	0.5
Y1Cl18C0.5	$(n\text{-}C_{18}H_{37})(CH_3)_2SiCl$	Aldrich		0.5
Y2Cl18C0.5	$(n\text{-}C_{18}H_{37})(CH_3)SiCl_2$	TCI	≥ 94	0.5
Y3Cl1C0.5	$(CH_3)SiCl_3$	Aldrich	99	0.5
Y3Cl1C10	$(CH_3)SiCl_3$	Aldrich		10.0
Y3Cl18C0.5	$(n\text{-}C_{18}H_{37})SiCl_3$	Aldrich	≥ 90	0.5

Tab. S.2: Textural properties of catalysts before and after the catalytic experiment, i.e., total specific surface area (A_{BET}), specific pore volume (V_{total}), difference in $A_{BET,}$ V_{total} compared to the fresh catalysts (ΔA_{BET}, ΔV_{total}).

Catalysts	Reducing agent	T / K	Before		After		Difference	
			A_{BET} a) / $m^2\ g^{-1}$	V_{total} b) / $cm^3\ g^{-1}$	A_{BET} a) / $m^2\ g^{-1}$	V_{total} b) / $cm^3\ g^{-1}$	ΔA_{BET} / %	ΔV_{total} / %
3PtY	FA	493	775	0.57	129	0.18	83	68
	H_2	493	775	0.57	123	0.29	84	49
	H_2	393	775	0.57	647	0.58	17	2
3PtY3Cl18C0.5	FA	493	483	0.37	44	0.11	91	70
	H_2	493	483	0.37	91	0.25	81	32
	H_2	393	483	0.37	276	0.31	43	16
3PtY3Cl1C10	FA	493	277	0.32	210	0.40	24	25

a) via BET. b) single point.
